# Revisiting The Relationship between The Ejaculatory Abstinence Period and Semen Characteristics

**DOI:** 10.22074/ijfs.2018.5192

**Published:** 2017-10-14

**Authors:** Bashir M Ayad, Gerhard Van der Horst, Stefan S Du Plessis

**Affiliations:** Division of Medical Physiology, Faculty of Medicine and Health Sciences, Stellenbosch University, Tygerberg, South Africa

**Keywords:** DNA Fragmentation, Semen Analysis, Sexual Abstinence, Spermatozoa, Sperm Motility

## Abstract

Variation in the ejaculatory abstinence period suggested by different guidance bodies have resulted in a growing concern among researchers and clinicians over what the precise period of ejaculatory abstinence ought to be for an optimal semen sample. Several studies have thus been undertaken to examine the association between the length of sexual abstinence and semen characteristics. Not all studies, however, have arrived at the same conclusions. This study aims to review all existing literature published during the past few decades pertaining to the influence of ejaculatory abstinence on semen quality. For the purpose of this systematic review, all data related to sexual abstinence duration and seminal parameters were re-analysed to homogenize the current data. Thorough PubMed, MEDLINE and Google Scholar, a literature search was conducted using the keywords “sexual abstinence”, “ejaculatory abstinence”, “semen”, “spermatozoa”, “semen analysis”, “sperm parameters”, “motility”, “reactive oxygen species (ROS)” and “DNA fragmentation”. After carefully reviewing all the literature, 30 relevant papers, both written in English and published between January 1979 and December 2016, were included in this review. The weight of the evidence suggests that the decline in semen volume and sperm concentration with shorter abstinence periods is accompanied by a substantial improvement in sperm motility characteristics, especially progressive motility and velocity. Nevertheless, available data are insufficient to support definitive conclusions regarding the influence of the ejaculatory abstinence period on advanced semen parameters (ROS, DNA fragmentation and seminal plasma antioxidant capacity) and pregnancy rates. In conclusion, taking all data into account, shortening of the abstinence period may be beneficial to sperm quality. Furthermore, we recommend that the current guidelines regarding the prescribed abstinence period should be revisited.

## Introduction

Infertility is the “failure to achieve a clinical pregnancy after 12 months or more of regular unprotected sexual intercourse” and is a condition estimated to affect about 15% of all couples of reproductive age. Male factor infertility has been found to be the sole contributor in approximately 20% of all infertility cases and is partially implicated in another 30-40% (1). When the attributable causes of female infertility have been eliminated and/or semen analysis results fail to meet the World Health Organization (WHO) criteria, male infertility is taken into consideration as the likely etiological factor. Therefore, semen analysis still remains the established cornerstone of the laboratory assessment of male infertility. 

A considerable amount of variability has been shown to exist in various semen characteristics within and among individuals (2). These variations have been largely attributed to several modifiable intrinsic and extrinsic factors. These factors include the length of sexual abstinence, ejaculation frequency and method of collection. Other factors that have the potential to influence semen quality are general health and lifestyle, infection, dysfunction of male sex glands, urogenital surgery as well as therapeutic and environmental exposures (3). 

The WHO manuals for examining and processing human semen provide a practical guide for standardizing semen analysis. These manuals have been periodically published and actively developed since its first edition in 1980. The WHO criteria for semen analysis have been adopted by most human andrology and fertility laboratories around the world for more than thirty years. The most recent guidelines of WHO recommend that the minimum period of ejaculatory abstinence prior to semen collection should not be less than 2 days and more than 7 days (4). The Nordic Association for Andrology (NAFA) and the European Society of Human Reproduction and Embryology (ESHRE) (5), however, outline a narrower range of 3-4 days of abstinence. The basis for these recommendations is nevertheless not supported by sufficient scientific evidence and requires further clarification. 

In light of the differing ejaculatory abstinence periods suggested by various regulatory bodies, a growing concern has resulted over what the precise period of ejaculatory abstinence ought to be for an optimal semen sample. This has prompted several studies to examine the influence of abstinence periods on various semen parameters. The results are, however, not conclusive. Interestingly, some studies have even challenged the recommended guidelines in favour of extremely shorter periods (i.e. <1 hour to 4 hours) due to their advantageous effects on semen characteristics (6-9). Studies on the association of abstinence length with semen quality have examined a wide range of abstinence intervals. Although numerous related articles have been published to this date, a systematic review has not been undertaken. This study therefore aims to review the existing scientific literature over the past few decades pertaining to this association in humans to evaluate the weight of evidence for the optimal time period of ejaculatory abstinence. 

## Materials and Methods

For the purpose of this systematic review, all data related to sexual abstinence duration and seminal parameters were re-analysed to homogenize the current data. An extensive review of the existing literature was performed in various electronic databases, namely MEDLINE, PubMed and Google Scholar by using the keywords “sexual abstinence”, “ejaculatory abstinence”, “semen”, “spermatozoa”, “semen analysis”, “sperm parameters”, “motility”, “reactive oxygen species (ROS)” and “DNA fragmentation”. A total of 34 relevant articles were obtained, all of which were written in English and published between January 1979 and December 2016. Four of these were excluded due to a lack of numerical data. After careful review of the abstracts, these 30 studies were included in the current review, of which 25 were prospective and five were retrospective. The majority of the included studies had used donors recruited from the general population, while twelve studies selected patients from infertility clinics or assisted reproduction units. The seminal parameters examined were semen pH, semen volume, sperm concentration, sperm motility, sperm morphology, sperm intracellular ROS and DNA fragmentation, and seminal plasma antioxidant capacity. Here, ejaculatory abstinence was classified into the time periods of ≤1 day, 2-3 days, 4-5 days, 6-7 days and ˃7 days. 

### Ejaculatory abstinence and conventional semen parameters

The majority of the studies investigating the influence of ejaculatory abstinence on semen quality (Table 1) had assessed the most conventional semen parameters (e.g. volume, count and concentration, motility and morphology) as described by WHO (4) with the latest version reporting a reference range based on men with proven fertility. 

### Seminal pH

A slightly alkaline seminal fluid is necessary to neutralize the acidic environment of the vagina, which can negatively impact sperm function (10). A substantial reduction in sperm motility was reported in patients with semen pH less than the WHO lower-bound threshold value of 7.2 (11), however, the correlation between semen pH and sperm motility was not statistically significant (12). Only three studies considered seminal pH as a parameter when investigating the relationship between the abstinence period and semen quality (13-15). Blackwell and Zaneveld (13) analysed semen samples from ten men with abstinence periods of 1, 2, 3, 4, 5 and 10 days, and found that seminal pH remained essentially unchanged. In addition, De Jonge et al. (14) examined ejaculates from 11 men who had abstained for 1, 3, 5 and 8 days, and reported no significant changes in seminal pH across the four abstinence periods. Similar results were also reported by Agarwal et al. (15) who collected semen samples from seven men each abstaining sequentially for 1, 2, 5, 7, 9 and 11 days, and observed that semen pH remained relatively stable but declined significantly after 11 days of abstinence. The scarcity of studies examining seminal pH indicates that the significance of this semen marker has been underestimated. 

### Semen volume

According to the latest WHO guidelines, the lower-bound reference value for semen volume is 1.5 ml. Accurate measurement of the ejaculate volume is important as the concentration of spermatozoa and non-sperm cells in the ejaculate are based on the initial volume. Semen volume-after the recommended standard period of abstinence-has consequently been suggested to be an early indicator of low semen quality even before identifying any abnormality in concentration, motility and morphology of spermatozoa. has also been suggested to be a reliable indicator of the secretory functions of the accessory glands, particularly the seminal vesicles (4). 

The relationship between the abstinence period and semen volume was reported in twenty-four studies (Table 1). In all but one, there is robust and consistent evidence for the significant increase in semen volume with increase in abstinence period (6,8,9,13,32). In a retrospective longitudinal study (22), the greatest overall mean of daily increase in semen volume was observed at 11.9% per day during the first 4 days of abstinence. However, only one study (33) failed to show any significant change in semen volume in both normozoospermic and asthenozoospermic populations which is likely to be due to the small sample size studied and the protracted period of the short abstinence. 

Short abstinence-associated decreases in the ejaculate volume may be attributed to insufficiency of the accessory sex glands to make an adequate contribution to the ejaculate volume, particularly the seminal vesicles and the prostate gland, which are the major contributors. The epithelial tissues of these organs are targeted by androgen, which is thought to regulate their mRNA production as well as the synthesis of rough endoplasmic reticulum, thereby enhancing the production of seminal plasma proteins (34). Improved secretory capacity of the seminal vesicles and the prostate gland has been associated with higher endogenous serum testosterone levels in rats (35) and men (36). In addition, higher testosterone serum levels have been reported following a prolonged abstinence period compared with a shorter abstinence (37). Therefore, the potential stimulating effect of testosterone on the major accessory glands associated with long abstinence periods may contribute to the increased semen volume after prolonged abstinence periods. 

### Sperm concentration and total count

Concentration of spermatozoa in semen, expressed as millions per millilitre, is a critical indicator of semen quality and a prognostic factor for fertility potential (38). However, it is not recommended as an accurate measure of spermatogenesis because it is influenced by the volume of secretions of the accessory sex glands in which the concentrated epididymal spermatozoa are diluted in during ejaculation (4). The total number of spermatozoa in the ejaculate, expressed as millions per total ejaculate and obtained by multiplying the sperm concentration by the semen volume, is suggested to be a better parameter for the evaluation of spermatogenic statuses (39). The lower-bound threshold values of sperm concentration and total count recommended by the WHO are 15×10^6^ spermatozoa/mL and 39×10^6^ spermatozoa/ejaculate respectively (4).

The influence of the abstinence period on sperm concentration was assessed in twenty-two of the studies listed in Table 1. Of these, twenty (91%) reported a linear increase in sperm concentration with increased abstinence periods (8, 9, 13-16, 19, 21-26, 28-30, 32, 33, 40, 41). The highest rise in the overall mean of sperm concentration (14×10^6^/mL) occurred when the abstinence period increased from 2-3 days to 4-5 days (Table 2). Two studies found a non-significant mild increase in sperm concentration after long abstinence compared with short abstinence periods (18, 20). Eighteen studies (6, 9, 13, 15, 16, 18, 19, 21-24, 29-33, 41, 42) reported a significant association between long abstinence periods and increased total sperm count in the ejaculate. The largest increase in the overall mean of total sperm count was recorded when the abstinence period extended from 6-7 days to ˃7 days.

During sexual inactivity an estimated 400 million spermatozoa are reserved within the epididymis with the majority stored in the cauda epididymis and lesser in the caput and corpora with an average of 90 million in each of these sections. The paired vas deferens with its ampulla is estimated to contain about 75 million spermatozoa (39). During the arousal phase, but prior to the emission phase, the population of spermatozoa in the paired ampulla increases dramatically as they move distally towards the urethra (43). After particularly long periods of abstinence, the bulk of the sperm population in the first ejaculate mainly comprise spermatozoa stored in the ampulla and vas deference, and partly in the cauda epididymis. Consequent ejaculates in quick successions are typically characterized by a lower total count of spermatozoa as the residual spermatozoa are flushed from the proximal cauda and corpus, and thereafter from the caput (6), all of which contain much lower sperm reserves (39). Despite these findings, Bahadur et al. (8) interestingly suggested that “combining the initial and consecutive ejaculates allows for a potential shift of severe and oligozoospermia patients towards the normospermia range”. This approach may lead to a change in the treatment strategies by possibly avoiding testicular biopsies.

The observed consistent positive correlation of sperm concentration and total count with increasing abstinence durations can be ascribed to daily sperm production, which is determined to be approximately 130-270×10^6^ per day (39). The regulation of testicular functions and spermatogenesis necessitates a complex combination of endocrine and paracrine signals. Relatively higher levels of testosterone are essential for the maintenance and proceeding of spermatogenesis. Serum testosterone levels were shown to fluctuate mainly from the second to the fifth day of abstinence, reaching a peak (about 145% of the baseline) after the seventh day of abstinence and remaining relatively constant even when the abstinence period was prolonged (37).

### Sperm motility and kinematics

Assessment of motility characteristics of ejaculated spermatozoa has been shown to have the utmost importance for the diagnosis of male fertility potential since it provides vital information on the functional competence of the spermatozoon. The percentage of motile spermatozoa in the ejaculate provides an indication of epididymal sperm maturation (44). However, progressive motility is required for the spermatozoa to migrate through the harsh environment of the female genital tract to reach the ovum. Motility is not only necessary for sperm transit, but changes in flagellar motion also play an essential role at the site of fertilization. The mechanical driving force generated by motility help the sperm to propel through the outer layers of the cumulusoocyte complex (45). The lower-bound WHO threshold values for the percentages of total motility and progressive motility are 40 and 32% respectively (4). 

Twelve studies examined the relationship between the abstinence period and the total motile sperm (TMS) count in the ejaculate. Eight of these (15,23,24,26,29,40,42) reported an increase in TMS count with increase in the abstinence period, while the other four did not find any significant effect of abstinence period on TMS (9,22,28,33). The overall mean of TMS increased substantially as the abstinence period increased from ≤ 1 to 3 days (Table 2). The mean TMS remained relatively stable between the fourth and the seventh day, increased on the subsequent days (>7) and declined gradually after day 9 to 10 of abstinence (17,19). The influence of abstinence length on the percentage of motile spermatozoa was investigated in seventeen studies (6,9,14,15,17,19,21,23,24,26,30,32,41,42) (Table 1). We found little consensus among the results of these studies. A slight or lack of association between abstinence period and motile sperm percentage was reported in eleven studies (9,14,15,17,21,27,28,30,32,41,42). In contrast, six studies (6,19,23,24,26,29) reported a substantial decrease in the percentage of motile spermatozoa with increasing abstinence; the highest overall mean sperm motility percentage was observed after ≤ 1 day of abstinence (Table 2). 

**Table 1:** Abstinence periods and semen characteristics


Type of study	Abstinence periods	Subjects	Number of subjects/samples	Volume (mL)	Concentration (10^6^/mL)	TSC (10^6^/ejaculate)	TMS (10^6^/ejaculate)	Motility (%)	Progressive motility (%)	Viability (%)	Normal morphology (%)	DNA fragmentation (%)	ROS

Prospective	4 hours and 3-5 days	Volunteers	11	↑	−−	↑	−−	↓	↓	−−	−−	−−	−−
Prospective	3 hours and 96 hours	Normozoospermic	21	−−	−−	−−	−−	−−	−−	−−	−−	↔	−−
Prospective	40 minutes and 2-7 days	Oligozoospermic	73	↑	↑	−−	−−	−−	↓	−−	↓	−−	−−
Prospective	2 hours and 3-4days	Healthy	3	↑	↑	↑	↔	↔	↔	↔	−−	↓	↔
Prospective	1, 2, 3, 4, 5 and 10 days	Volunteers	10	↑	↑	↑	−−	−−	−−	↔	↑	−−	−−
Prospective	1, 3, 5, and 8 days	Volunteers	11	↑	↑	−−	−−	↔	−−	↔	↔	↔	−−
Prospective	1, 2, 5, 7, 9 and 11 days	Normozoospermic	7	↑	↑	↑	↑	↔	−−	↓	−−	↑	↔
Prospective	1, 2, 3, 4, 5, 6 and 7 days	Normal	36	↑ ↑	↑	↑	−−	−−	−−	−−	−−	−−	−−
Retrospective	≤1, 2, 3, 4, 5, 6 and 7 days	Suspected infertile	1801	↑	−−	−−	−−	↔	−−	↔	↔	−−	−−
Prospective	8 hours and 3 days	Volunteers	7	↑	↔	↑	−−	−−	−−	−−	↔	−−	−−
Prospective	12 hours and 7 days	Volunteers	10	↑	↑	↑	−−	↓	−−	−−	↔	−−	−−
Prospective	2-4, 5-7 and ˃7 days	Healthy men	195	↑	↔	−−	−−	−−	−−	−−	−−	−−	−−
Prospective	2, 4, 7, 10, 15 and 18 days	Volunteers	6	↑	↑	↑	−−	↔	−−	−−	↔	−−	−−
Prospective	˂4, 4-6 and ˃6 days	Healthy	27	↑	↑	↑	↔	−−	−−	−−	↔	−−	−−
Prospective	2-3 and 4-7 days	Non-azoospermic	422	↑	↑	↑	↑	↓	↓	−−	↓	−−	−−
Retrospective	1, 2, 3, 4, 5, 6, 7, 8-10 and 11-14 days	Oligozoospermic	3506samples	↑	↑	↑	↑	↓	−−	−−	↓	−−	−−
Retrospective	1, 2, 3, 4, 5, 6, 7, 8-10 and 11-14 days	Normozoospermic	5983 samples	↑	↑	↑	↑	↓	−−	−−	↔	−−	−−
Prospective	2, 3, 4 and 5 days	Fertile	500	↑	↑	−−	−−	−−	↓	−−	↔	−−	−−
Retrospective	≤2 and 3-7 days	Undergoing IUI	372	↑	↑	−−	↑	↓	−−	↓	−−	−−	−−
Prospective	1 and 4 days	Undergoing ICSI	40	↑	−−	−−	−−	↔	−−	−−	−−	↑	−−
Prospective	18-30 hours and 3-5 days	Healthy	57	↑	↑	−−	↔	↔	−−	−−	↑	↑	−−
Prospective	1, 2, 3, 4, 5, 6, 7, 8, 9 and 10 days	Normozoospermic	100 samples	↑	↑	↑	↑	↓	−−	↔	↔	−−	−−
Prospective	1 and 4 days	Planning IUI	40	↑	↑	↑	−−	↔	−−	−−	↔	−−	−−
Retrospective	2-3, 4-5 and 6-7 days	Attending Infertility Unit	730	↑	−−	↑	−−	−−	↓	↓	↔	−−	−−
Prospective	1 and 3-4 days	Healthy	6	↑	↑	↑	−−	↔	↔	↔	↔	↔	↔
Prospective	3, 6 and 10 days	Normozoospermic	7	↔	↑	↑	↔	−−	↓	↔	↔	−−	−−
Prospective	3, 6 and 10 days	Asthenozoospermic	7	↔	↑	↑	↔	−−	↔	↔	↔	−−	−−
Retrospective	≤3, 4-10 and ˃10 days	Undergoing IUI	929	−−	↑	−−	↑	−−	↔	−−	−−	−−	−−
Prospective	4 and 14 days	Nonobstructive azoospermic	50	−−	↑	↑	↑	↔	−−	−−	−−	−−	−−
Prospective	1, 2, 4, 7, 10 and 14 days	Healthy	4	−−	−−	↑	↑	↔	↔	↔	↔	−−	−−
Prospective	1 and 3-4 days	With high DNAfragmentation levels	35	−−	−−	−−	−−	−−	−−	−−	−−	↑	−−
Prospective	1-10 days	Healthy	36 samples	−−	−−	−−	−−	−−	−−	−−	−−	−−	↔


TSC; Total sperm count/ejaculate, TMS; Total motile sperm/ejaculate, ROS; Reactive oxygen species, ICSI; Intracytoplasmic sperm injection, IUI; Intrauterine insemination, ↑; Increase significantly with increasing abstinence period (P≤0.05), ↓; Decrease significantly with increasing abstinence period (P≤0.05), ↔; Not significantly different, and --; Not investigated.

**Table 2:** The overall mean values of basic semen parameters in relation to different abstinence periods calculated from values reported in relevant studies referred to in Table 1


Semen parameter	Day				
	≤1	2-3	4 -5	6-7	˃7

Semen volume (mL)	2.198	2.72	3.251	3.773	4.229
	n=15	n=13	n=18	n=13	n=14
Concentration (10^6^/mL)	54.363	52.038	66.849	64.623	70.474
	n=16	n=11	n=16	n=11	n=13
Total sperm count (10^6^/ejaculate)	99.911	114.306	172.591	225.792	288.642
	n=9	n=9	n=10	n=10	n=12
Total motile sperm (10^6^/ejaculate)	36.56	49.618	81.114	78.517	94.612
	n=8	n= 8	n=9	n=8	n=9
Motility (%)	56.03	44.813	52.044	41.277	43.325
	n=15	n=11	n=15	n=12	n=13
Progressive motility (%)	57.083n=6	54.533n=3	53.887	49.15	-
			n=6	n=2	-
Viability (%)	66.29	72.37	73.622	68.4	66.41
	n=4	n=5	n=5	n=5	n=6
Normal morphology (%)	8.453	9.644	10.16	8.45	8.590
	n=1	n=14	n=15	n=14	n=13


The average reported in each study contributed equally to the overall mean. Only studies reporting absolute values were included. All studies were included in the calculations (e.g. normozoospermic, oligozoospermic, volunteers, patients, etc.).

Ten studies (6,8,9,23,25,31,33,40,42) investigated the relationship between ejaculatory abstinence and progressive motility (Table 1). Five studies (6,8,23,25,31) reported a significantly higher percentage of progressively motile spermatozoa with shorter abstinence periods, with the overall mean peak of progressive motility observed after ≤1 day of abstinence (Table 2). Interestingly, shortening the abstinence interval to about 30 minutes resulted in a significant increase in the percentage of fast progressive (type A) spermatozoa (8). The results of Magnus et al. (33) were consistent with those of the abovementioned studies where the progressive motility of a normozoospermic population was found to increase with decreasing abstinence time. However, they analysed an asthenozoospermic population and found no such association, corroborating the findings of the other relevant studies (9,32,40,42). 

Motility assessment in the majority of the studies was performed manually using a light microscope and only five studies (23, 27-29, 42) used computer-aided sperm analysis (CASA). Manual assessment of sperm motility is subjective and is strongly associated with inter- and intra-laboratory variation (46). The potential counting and interpretation errors associated with the subjective visual assessment of sperm motility have made automated semen analyses an absolute necessity. CASA, in contrast to subjective motility estimation, is certainly a powerful approach for the objective assessment of sperm motion. The most recent WHO guidelines on semen analysis nevertheless indicate that the assessment of sperm motility percentage using CASA may be unreliable due to the potential misidentification of particulate debris as immotile spermatozoa (4). This issue has recently been addressed as modern CASA systems such as the sperm class analyser (SCA6) are now equipped with intelligent filters to accurately identify the spermatozoa and eliminate the debris and other cells. The automatic analysis of sperm motility by CASA instruments enables the objective estimation of various parameters which translate into certain kinematic measures of sperm movement (47). The only study investigating the impact of ejaculatory abstinence on sperm kinematics, among other determinants of semen quality, had been conducted by Elzanaty et al. (23). In this study, semen samples collected from patients with a wide age range, undergoing infertility assessment, were grouped into three categories based on the abstinence period (i.e. 2-3 days, 4-5 days and 6-7 days). Significantly higher straight-line velocity (VSL) and linearity (LIN) were found in the group with the shortest abstinence period, while average path velocity (VAP) and curvilinear velocity (VCL) were not significantly different among the three abstinence groups.

Variation in semen characteristics among individuals may enhance the potential for observation bias (48) since other factors besides ejaculatory abstinence may account for the effects observed. However, collecting replicate semen samples from the same individual is likely to be an effective approach to controlling confounding factors. The increase in semen volume and sperm concentration with prolonged abstinence periods was almost consistently accompanied by substantial deterioration in sperm motility characteristics, especially progressive motility and velocity. Although the exact mechanism as to how ejaculatory abstinence may affect changes in semen quality is unknown, a number of possibilities have been suggested. For instance, reduction in the storage period within the epididymis may minimize the exposure of unejaculated spermatozoa to motility inhibitory factors and enzymes released from the degenerating cells within the same microenvironment (6). Furthermore, the sperm reservoir capacity of the cauda epididymis is limited (49), thus the substantial increase in sperm concentration during prolonged ejaculatory abstinence may result in the depletion of energy reserves and allow for senescent spermatozoa to accumulate in the epididymis. The relative contribution of these senescent spermatozoa to the subsequent ejaculate impairs semen quality (27, 50). Extending the abstinence time may also enhance susceptibility of unejaculated spermatozoa to recurrent genital heat exposure, causing detrimental changes to the membrane phospholipid architecture of epididymal spermatozoa (51) and the functional properties of the motor apparatus of the sperm flagellum (52). Therefore, reducing the abstinence period may minimize the frequency and time span of heat exposure, thereby leading to improved motility.

### Sperm viability

Sperm viability is one of the parameters that is routinely assessed in basic semen analysis, and is especially recommended in samples where the percentage of motile spermatozoa is less than about 40% (4). The viability status of spermatozoa selected for intracytoplasmic sperm injection (ICSI) has to be precisely examined since the injection of a live spermatozoon is vital to the success of the ICSI outcome (53). Furthermore, a direct correlation has recently been identified between sperm viability and the level of DNA fragmentation, showing that the viability status may be a potential indicator of DNA integrity of the ejaculated spermatozoa (54). The lowerbound reference limit for sperm viability is estimated to be 58% (4). The influence of abstinence duration on sperm viability was examined in eleven studies (9,13,15,17,26,29,31,33,42). This was done by using various techniques including a dye exclusion assay (14,15,29,33), the hypo-osmotic swelling test (13,31,42) and flow cytometry (9,32). Most of these studies reported slight or no statistically significant negative association between sperm viability and abstinence period. The overall mean percentage of viable spermatozoa peaked and remained relatively unchanged between the second and the fifth day of abstinence, and declined thereafter (Table 2). 

### Sperm morphology

To be considered morphologically normal, the whole spermatozoon and its three distinct areas, the head, midpiece and the tail, must fit with stringent criteria in terms of their size and shape. The 5th centile lower-bound reference limit for normal forms is 4% (4). It has also been reported that morphologically abnormal spermatozoa, with a special focus on the acrosomal region, have a lower chance to bind to the zona pellucida (55). A correlation has also been observed between sperm head abnormalities and DNA integrity. Therefore, analysis of sperm morphology, which may provide crucial evidence about semen quality, is assessed by fairly simple and inexpensive methods compared with expensive and elaborate assays such as DNA fragmentation (56) and acrosome reaction (57). The relationship between the abstinence duration and sperm morphology was investigated in eighteen studies (8,13,14,17,19,21,25,28,33,42). All had assessed sperm morphology manually via visual assessment except one (29) which had used CASA. 

Most of the studies (14 out of 18) reported no significant association between sperm morphology and the period of abstinence. In contrast, one study reported significantly higher percentages of spermatozoa with tail defects when the abstinence period was extended from 2-3 days to 6-7 days. However, the overall proportion of normal morphology did not differ between the two abstinence groups (23). Furthermore, Levitas et al. (24) reported that among mild to moderate oligozoospermic samples, the highest percentage of normal morphology was reported at ≤2 days of abstinence but this association was not observed in a normozoospermic population. Bahadur et al. (8) recently reported that an extremely short abstinence period of 30 minutes could significantly improve sperm morphology among oligozoospermic men, all candidates for intrauterine insemination (IUI) treatment. By contrast, shortening the abstinence duration in normal individuals from 3-5 days to only 18-30 hours resulted in a considerably lower percentage of morphologically normal spermatozoa (28). It may therefore be advantageous for patients with oligozoospermia to abstain for shorter periods before sperm collection in the process of fertility treatment. However, it must be re-iterated that manual assessment of sperm morphology is a subjective analysis with interand intralaboratory variation. This variability may be attributed to several factors including the use of different fixation and staining techniques (58), differences in interpretation (59) and technician expertise (60). Another important factor that needs to be taken into consideration is that the WHO guidelines and reference ranges have changed over the years and may thus lead to differences in interpretation (4). 

### Ejaculatory abstinence and advanced semen parameters

Conventional semen parameters provide the essential information on which clinicians base their preliminary diagnosis (61). Approximately 25-40% of idiopathic infertile males have been reported to have normal semen profiles (62). Therefore, a range of advanced sperm quality parameters have been developed to circumvent the limitations of the conventional semen analysis (63). 

### DNA fragmentation

Assessment of sperm DNA integrity, in addition to routine semen analysis, provides further valuable information about sperm quality as well as pregnancy outcomes (64,65). It has been shown that high proportions of spermatozoa with DNA fragmentation above 20% increase the risk of infertility regardless of having normal basic semen parameters (61). Eight studies (7,9,14,15,27,28,32,66) had investigated the relationship between the abstinence period and sperm DNA fragmentation. Three studies (7,14,32) did not find any effect while half of the studies (15, 27, Ayad et al. 28, 66) showed an increase in sperm DNA fragmentation rates with prolonged abstinence. Interestingly, the report by Mayorga-Torres et al. (9) was to the contrary, showing considerable increase in DNA fragmentation levels after an extremely short abstinence periods of 2 hours compared with the initial ejaculate that was collected after 3-4 days of abstinence. The latter finding could be purely a result of the extremely small and underrepresented sample size (n=3) but still merits further investigation. 

### Reactive oxygen species production

Normal physiological levels of ROS are crucial for maintaining various vital functions in spermatogenesis at different maturational stages. These highly reactive species can also act as essential mediators for signal transduction involved in sperm capacitation, hyperactivation and acrosome reaction (67). However, ROS levels must be maintained within physiological ranges since ROS overproduction or insufficient antioxidant defense can result in a state of oxidative stress (68). 

Three studies (9,15,32) examined the relationship between the abstinence period and sperm intracellular ROS production, while only one study examined the relationship in terms of seminal ROS concentration (69). These studies consistently reported no association of abstinence duration with either intracellular ROS production or seminal ROS levels. However, among the relevant studies a general trend of reduction, albeit non-significant, was observed in intracellular ROS levels after short abstinence in comparison with long abstinence. Interestingly, when four repeated ejaculates were collected on the same day at 2 hour intervals, a significant reduction in intracellular ROS production was observed in the fourth ejaculate compared with the initial one obtained after 3 to 4 days of abstinence (9). During their maturation and storage, spermatozoa are continuously susceptible to oxidative damage induced by intracellular and extracellular reactive species. Spermatozoa are highly sensitive to ROS damage by lipid peroxidation due to their membranes being highly rich in polyunsaturated fatty acids (67). Therefore, the release of spermatozoa through more frequent ejaculations may possibly minimize their adverse effects on sperm quality (9). 

### Seminal plasma antioxidants

Spermatozoa have limited intracellular enzymatic defense against oxidative stress, partly due to cytoplasmic extrusion during spermatogenesis. This deficient capacity is effectively compensated for by a group of cellular detoxifying enzymes with powerful antioxidant properties including superoxide dismutase (SOD), catalase (CAT), and glutathione peroxidises found within the seminal plasma (68). Surprisingly, only one study had examined the influence of ejaculatory abstinence period on seminal plasma antioxidants and lipid peroxidation of the sperm membrane (30). By analysing ejaculates of forty men undergoing IUI, Marshburn et al. (30) observed a significant improvement in the total antioxidant capacity of seminal plasma after one day of abstinence compared to four days. Lipid peroxidation of the sperm membrane remained unchanged between the two abstinence periods. They therefore suggested that short abstinence-related increase of total antioxidant capacity in seminal plasma could defend spermatozoa against oxidative stress through a mechanism that is independent of lipid peroxidation. Hitherto, there are no available data on the effect of the abstinence period on acrosome reaction or any individual antioxidants. With respect to the detoxyifying enzymes, however, we have observed in our laboratory that a short abstinence period of four hours led to a significant increase in SOD activity but did not change the activity of catalase in seminal plasma (unpublished data). 

### Pregnancy rate

The conventional parameters of semen analysis provide fundamental information for the initial diagnosis of male infertility, but none is reliable enough to predict pregnancy (38). Few studies had examined the influence of ejaculatory abstinence period on pregnancy rate, all of which had recruited patients from infertility and assisted conception clinics. For instance, among infertile couples undergoing ovulation induction followed by IUI, the highest pregnancy rate was observed for those with an abstinence period of ≤3 days, while a sharp decline in pregnancy rate was observed for those with ≥10 days of abstinence. Interestingly, the relationship between ejaculatory abstinence period and pregnancy rate was independent of the variation in conventional semen parameters (40). Another study, examining a more general infertile population, revealed that the highest IUI pregnancy rates were associated with ≤2 days of abstinence (26). Sánchez-Martín et al. (27) reported that serial ejaculation every 24 hours for four days with an ultimate abstinence of 12 hours, along with sperm selection by density gradient centrifugation, could significantly improve pregnancy rate with ICSI. More recently, Bahadur et al. (70) showed in a pilot study that recurrent ejaculates successfully improved IUI pregnancy rates. These findings can be supported by the fact that fertilization rates are directly related to sperm progressive motility and inversely related to DNA fragmentation *in vitro *(71) with both parameters generally found to be improved with shorter abstinence periods. However, importantly, large prospective randomized controlled trials are required to validate that short abstinence periods improve pregnancy and live birth rates, and may thus be recommended for infertility treatments. 

## Conclusion

We conclude that in spite of the varied quality of existing studies, the weight of evidence suggests that reducing the ejaculatory abstinence period may positively influence semen quality based on a consistent trend towards an increase in the percentage of motile, progressively motile and rapid spermatozoa with shorter abstinence periods. However, the small number of studies examining ROS production, DNA fragmentation and seminal plasma antioxidant capacity limit any definitive conclusion regarding its effect on advanced semen parameters. Further clinical trials with sufficient number of subjects, and controlling for potential confounders, may shed further light on this association. We recommend that future studies incorporate CASA as a more accurate and objective measurement tool as well as utilize more sensitive measures of sperm function such as sperm hyperactivity, sperm-zona binding ability, acrosome reaction, and total and individual seminal plasma antioxidants. It is, however, worth mentioning that even after short abstinence periods of ≤1 day, the overall mean values of the conventional semen parameters were always above the lower-bound reference limits recommended by WHO (fifth version). Therefore, shortening the abstinence period may be a potential strategy to improve sperm quality. It is thus recommended that the current guidelines regarding the prescribed abstinence period are revisited. 

## References

[1] Sharlip ID, Jarow JP, Belker AM, Lipshultz LI, Sigman M, Thomas AJ (2002). Best practice policies for male infertility. Fertil Steril.

[2] Alvarez C, Castilla JA, Martinez L, Ramírez JP, Vergara F, Gaforio JJ (2003). Biological variation of seminal parameters in healthy subjects. Hum Reprod.

[3] Du Plessis SS, Agarwal A, Sabanegh Jr ES (2014). Male infertility: a complete guide to lifestyle and environmental factors.

[4] World Health Organization (2010). WHO laboratory manual for the examination and processing of human semen.

[5] Kvist U, Björndahl L (2002). Manual on basic semen analysis.ESHRE Monographs.

[6] Valsa J, Skandhan KP, Gusani PH, Sahab Khan P, Amith S (2013). Quality of 4‐hourly ejaculates-levels of calcium and magnesium. Andrologia.

[7] Gosálvez J, González-Martínez M, López-Fernández C, Fernández JL, Sánchez-Martín P (2011). Shorter abstinence decreases sperm deoxyribonucleic acid fragmentation in ejaculate. Fertil Steril.

[8] Bahadur G, Almossawi O, Zeirideen Zaid R, Ilahibuccus A, Al-Habib A, Muneer A (2016). Semen characteristics in consecutive ejaculates with short abstinence in subfertile males. Reprod Biomed Online.

[9] Mayorga-Torres JM, Agarwal A, Roychoudhury S, Cadavid A, Cardona-Maya  DW (2016). Can a short term of repeated ejaculations affect seminal parameters?. J Reprod Infertil.

[10] Nakano FY, Leão RD, Esteves SC (2015). Insights into the role of cervical mucus and vaginal pH in unexplained infertility. MedicalExpress.

[11] Zhou J, Chen L, Li J, Li H, Hong Z, Xie M (2015). The semen PH affects sperm motility and capacitation. PLoS One.

[12] Natarajamani S, Janani D, Subramanian M, Manikere A (2014). Correlation of semen PH with other semen parameters in a sub fertile male population attending a tertiary ART center in South India. International Journal of Scientific and Research Publication.

[13] Blackwell JM, Zaneveld LJ (1992). Effect of abstinence on sperm acrosin, hypoosmotic swelling, and other semen variables. Fertil Steril.

[14] De Jonge C, LaFromboise M, Bosmans E, Ombelet W, Cox A, Nijs M (2004). Influence of the abstinence period on human sperm quality. Fertil Steril.

[15] Agarwal A, Gupta S, Du Plessis S, Sharma R, Esteves SC, Cirenza C (2016). Abstinence time and its impact on basic and advanced semen parameters. Urology.

[16] Schwartz D, Laplanche A, Jouannet P, David G (1979). Within-subject variability of human semen in regard to sperm count, volume, total number of spermatozoa and length of abstinence. J Reprod Fertily.

[17] Mortimer D, Templeton AA, Lenton EA, Coleman RA (1982). Influence of abstinence and ejaculation-to-analysis delay on semen analysis parameters of suspected infertile men. Arch Androl.

[18] Oldereid NB, Gordeladze JO, Kirkhus B, Purvis K (1984). Human sperm characteristics during frequent ejaculation. J Reprod Fertil.

[19] Le Lannou D, Colleu D, Boujard D, Le Couteux A, Lescoat D, Segalen J (1986). Effect of duration of abstinence on maturity of human spermatozoa nucleus. Arch Androl.

[20] Padova G, Tita P, Briguglia G, Giuffrida D (1988). Influence of abstinence length on ejaculate characteristics. Acta Eur Fertil.

[21] Pellestor F, Girardet A, Andreo B (1994). Effect of long abstinence periods on human sperm quality. Int J Fertil Menopausal Stud.

[22] Carlsen E, Petersen JH, Andersson AM, Skakkebaek NE (2004). Effects of ejaculatory frequency and season on variations in semen quality. Fertil Steril.

[23] Elzanaty S, Malm J, Giwercman A (2005). Duration of sexual abstinence: epididymal and accessory sex gland secretions and their relationship to sperm motility. Hum Reprod.

[24] Levitas E, Lunenfeld E, Weiss N, Friger M, Har-Vardi I, Koifman A (2005). Relationship between the duration of sexual abstinence and semen quality: analysis of 9,489 semen samples. Fertil Steril.

[25] Sobreiro BP, Lucon AM, Pasqualotto FF, Hallak J, Athayde KS, Arap S (2005). Semen analysis in fertile patients undergoing vasectomy: reference values and variations according to age, length of sexual abstinence, seasonality, smoking habits and caffeine intake. Sao Paulo Med J.

[26] Marshburn PB, Alanis M, Matthews ML, Usadi R, Papadakis MH, Kullstam S (2010). A short period of ejaculatory abstinence before intrauterine insemination is associated with higher pregnancy rates. Fertil Steril.

[27] Sánchez-Martín P, Sánchez-Martín F, González-Martínez M, Gosálvez J (2013). Increased pregnancy after reduced male abstinence. Syst Biol Reprod Med.

[28] Sukprasert M, Wongkularb A, Rattanasiri S, Choktanasiri W, Satirapod C (2013). The effects of short abstinence time on sperm motility, morphology and DNA mDamage. Andrology.

[29] Choavaratana R, Jirattigalachote A, Phornwilardsiri S, Thokha B, Orachon D (2014). Effect of abstinence duration on sperm sex ratio. Siriraj Med J.

[30] Marshburn PB, Giddings A, Causby S, Matthews ML, Usadi RS, Steuerwald N (2014). Influence of ejaculatory abstinence on seminal total antioxidant capacity and sperm membrane lipid peroxidation. Fertil Steril.

[31] Sunanda P, Panda B, Dash C, Padhy RN, Routray P (2014). Effect of age and abstinence on semen quality: A retrospective study in a teaching hospital. Asian Pac J Reprod.

[32] Mayorga-Torres BJ, Camargo M, Agarwal A, du Plessis SS, Cadavid ÁP, Cardona Maya WD (2015). Influence of ejaculation frequency on seminal parameters. Reprod Biol Endocrinol.

[33] Magnus O, Tollefsrud A, Abyholm T, Purvis K (1991). Effects of varying the abstinence period in the same individuals on sperm quality. Arch Androl.

[34] Fawell SE, Higgins SJ (1984). Androgen regulation of specific mRNAs, endoplasmic reticulum and Golgi-system. Mol Cell Endocrinol.

[35] Zanato VF, Martins MP, Anselmo-Franci JA, Petenusci SO, Lamano-Carvalho TL (1994). Sexual development of male Wistar rats. Braz J Med Biol Res.

[36] Gonzales GF (2002). Basal serum testosterone as an indicator of response to clomiphene treatment in human epididymis, seminal vesicles and prostate. Andrologia.

[37] Jiang M, Jiang J, Zou Q, Shen JW (2003). A research on the relationship between ejaculation and serum testosterone level in men. J Zhejiang Univ Sci.

[38] Guzick DS, Overstreet JW, Factor-Litvak P, Brazil CK, Nakajima ST, Coutifaris C (2001). Sperm morphology, motility, and concentration in fertile and infertile men. N Engl J Med.

[39] Amann RP (2009). Evaluating spermatogenesis using semen: the biology of emission tells why reporting total sperm per sample is important, and why reporting only number of sperm per milliliter is irrational. J Androl.

[40] Jurema MW, Vieira AD, Bankowski B, Petrella C, Zhao Y, Wallach E (2005). Effect of ejaculatory abstinence period on the pregnancy rate after intrauterine insemination. Fertil Steril.

[41] Raziel A, Friedler S, Schachter M, Kaufman S, Omanski A, Soffer Y (2001). Influence of a short or long abstinence period on semen parameters in the ejaculate of patients with nonobstructive azoospermia. Fertil Steril.

[42] Cooper TG, Keck C, Oberdieck U, Nieschlag E (1993). Effects of multiple ejaculations after extended periods of sexual abstinence on total, motile and normal sperm numbers, as well as accessory gland secretions, from healthy normal and oligozoospermic men. Hum Reprod.

[43] Durairajanayagam D, Rengan AK, Sharma RK, Agarwal A, Schattman GL, Esteves S, Agarwal A (2015). Sperm biology from production to ejaculation. Unexplained infertility: pathophysiology, evaluation and treatment.

[44] Fàbrega A, Puigmulé M, Bonet S, Pinart E (2012). Epididymal maturation and ejaculation are key events for further in vitro capacitation of boar spermatozoa. Theriogenology.

[45] Suarez SS, Pacey AA (2006). Sperm transport in the female reproductive tract. Hum Reprod Update.

[46] Cooper TG, Yeung CH (2006). Computer-aided evaluation of assessment of “grade a” spermatozoa by experienced technicians. Fertil Steril.

[47] Mortimer ST, van der Horst G, Mortimer D (2015). The future of computeraided sperm analysis. Asian J Androl.

[48] Keel BA (2006). Within-and between-subject variation in semen parameters in infertile men and normal semen donors. Fertil Steril.

[49] Bedford JM (1994). The status and the state of the human epididymis. Hum Reprod.

[50] Mortimer D (1994). Practical laboratory andrology.

[51] Wechalekar H, Setchell BP, Peirce EJ, Ricci M, Leigh C, Breed WG (2010). Whole-body heat exposure induces membrane changes in spermatozoa from the cauda epididymidis of laboratory mice. Asian J Androl.

[52] Saikhun J, Kitiyanant Y, Vanadurongwan V, Pavasuthipaisit K (1998). Effects of sauna on sperm movement characteristics of normal men measured by computer-assisted sperm analysis. Int J Androl.

[53] Nagy ZP, Liu J, Joris H, Verheyen G, Tournaye H, Camus M (1995). The result of intracytoplasmic sperm injection is not related to any of the three basic sperm parameters. Hum Reprod.

[54] Samplaski MK, Dimitromanolakis A, Lo KC, Grober ED, Mullen B, Garbens A (2015). The relationship between sperm viability and DNA fragmentation rates. Reprod Biol Endocrinol.

[55] Garrett C, Liu DY, Baker HW (1997). Selectivity of the human sperm-zona pellucida binding process to sperm head morphometry. Fertil Steril.

[56] Zini A, Phillips S, Courchesne A, Boman JM, Baazeem A, Bissonnette F (2009). Sperm head morphology is related to high deoxyribonucleic acid stainability assessed by sperm chromatin structure assay. Fertil Steril.

[57] Menkveld R, El-Garem Y, Schill WB, Henkel R (2003). Relationship between human sperm morphology and acrosomal function. J Assist Reprod Genet.

[58] Maree L, Du Plessis SS, Menkveld R, van der Horst G (2010). Morphometric dimensions of the human sperm head depend on the staining method used. Hum Reprod.

[59] Kruger TF, Coetzee K (1999). The role of sperm morphology in assisted reproduction. Hum Reprod Update.

[60] Barroso G, Mercan R, Ozgur K, Morshedi M, Kolm P, Coetzee K (1999). Intra-and inter-laboratory variability in the assessment of sperm morphology by strict criteria: impact of semen preparation, staining techniques and manual versus computerized analysis. Hum Reprod.

[61] Giwercman A, Lindstedt L, Larsson M, Bungum M, Spano M, Levine RJ (2010). Sperm chromatin structure assay as an independent predictor of fertility in vivo: a case-control study. Int J Androl.

[62] Gudeloglu A, Brahmbhatt J, Parekattil S, Glenn L (2015). Definitions and epidemiology of unexplained male infertility. Unexplained infertility.

[63] Erenpreiss J, Spano M, Erenpreisa J, Bungum M, Giwercman A (2006). Sperm chromatin structure and male fertility: biological and clinical aspects. Asian J Androl.

[64] Tesarik J, Greco E, Mendoza C (2004). Late, but not early, paternal effect on human embryo development is related to sperm DNA fragmentation. Hum Reprod.

[65] Borini A, Tarozzi N, Bizzaro D, Bonu MA, Fava L, Flamigni C (2006). Sperm DNA fragmentation: paternal effect on early post-implantation embryo development in ART. Hum Reprod.

[66] Pons I, Cercas R, Villas C, Braña C, Fernández-Shaw S (2013). One abstinence day decreases sperm DNA fragmentation in 90% of selected patients. J Assist Reprod Genet.

[67] Du Plessis SS, Agarwal A, Halabi J, Tvrda E (2015). Contemporary evidence on the physiological role of reactive oxygen species in human sperm function. J Assist Reprod Genet.

[68] Du Plessis SS, McAllister DA, Luu A, Savia J, Agarwal A, Lampiao F (2010). Effects of H(2)O(2) exposure on human sperm motility parameters, reactive oxygen species levels and nitric oxide levels. Andrologia.

[69] Desai NR, Mahfouz R, Sharma R, Gupta S, Agarwal A (2010). Reactive oxygen species levels are independent of sperm concentration, motility, and abstinence in a normal, healthy, proven fertile man: a longitudinal study. Fertil Steril.

[70] Bahadur G, Almossawi O, Illahibuccus A, Okolo S (2016). Factors leading to pregnancies in stimulated intrauterine insemination cycles and the use of consecutive ejaculations within a small clinic environment. J Obstet Gynaecol India.

[71] Simon L, Lewis SE (2011). Sperm DNA damage or progressive motility: which one is the better predictor of fertilization in vitro. Syst Biol Reprod Med.

